# European Identity in Youth: Longitudinal Dynamics and the Role of School Experiences

**DOI:** 10.1002/jad.70097

**Published:** 2026-01-13

**Authors:** Astrid Körner, Katharina Eckstein, Anna‐Maria Mayer, Philipp Jugert, Peter Noack

**Affiliations:** ^1^ Friedrich Schiller University Jena Jena Germany; ^2^ Catholic University Eichstätt‐Ingolstadt Eichstätt Germany; ^3^ University of Duisburg‐Essen Essen Germany

**Keywords:** adolescence, European identity, latent transition analysis, school, solidarity

## Abstract

**Introduction:**

For young people in Europe, European identity can serve as an important source of solidarity and belonging, especially in times of growing societal polarization. This study investigates European identity development during adolescence with two aims: (1) to identify European identity profiles, their associations with civic and solidarity‐related attitudes, and profile changes over time; and (2) to examine the role of school‐based experiences in predicting profile membership and transitions.

**Methods:**

Drawing on longitudinal data from German 9th graders collected at the beginning and end of one school year (*N *= 1,206; *M*Age = 14.39 years; 51.7% female), Latent Profile Analysis (LPA) and Latent Transition Analysis (LTA) were used to examine stability and change of European identity profiles. Based on recent process‐oriented models, European identity captured the processes of commitment, in‐depth exploration, and reconsideration. Civic and solidarity‐related correlates of status profile encompassed EU‐related attitudes, tolerance, and intentions for civic engagement; school‐based predictors included students' supportive relationships and pluralistic learning climate.

**Results:**

Analyses revealed four distinct profiles reflecting different levels of identity consolidation, meaningfully associated with civic‐ and solidarity‐related attitudes (i.e., tolerance, intentions for civic engagement). A more pluralistic climate was associated with more elaborate identity profiles at the beginning of the school year, while supportive student–teacher relationships were linked to forms of early closure. Yet, school experiences hardly predicted profile change across time.

**Conclusions:**

The findings underscore adolescence as a formative period for developing European identity and highlight both the potential and limitations of schools in supporting youth identity formation.

A European identity is considered essential for fostering European support, as well as promoting collective belonging, shared values, and cultural connectedness (Ciaglia et al. 2018; Hooghe and Marks 2004). For young people in Europe in particular, it is a crucial source of solidarity as they navigate their identity formation in challenging times, marked by crises such as the war in Ukraine and the rise of populist and anti‐democratic movements around the globe. These dynamics drive societal polarization, highlighting both the urgency and the difficulty of fostering a collective sense of belonging. Understanding how a European identity emerges and which factors shape this development is therefore of particular significance. To date, longitudinal studies on stability and change of identities beyond the personal domain (e.g., regional or political identities), as well as on the role of experiences in youth's immediate surroundings on their identity formation remain scarce. Building on recent process‐oriented models of identity status (e.g., Crocetti et al. 2008; Luyckx et al. 2008), this study examines the stability and change of European identity in adolescence and its association to civic‐ and solidarity‐related attitudes. Given the importance of social context for identity development (Lannegrand‐Willems and Bosma 2006; Sabatier 2008), the study also aims at shedding light on the role of school‐based experiences for the formation of European identity among adolescents.

## 1 European Identity Development: Identity Statuses and Their Role in Social and Civic Solidarity

Erikson (1959) described identity development as an “identity crisis” that primarily occurs during adolescence. If successfully resolved, adolescents manage to move from an initial uncertainty and diffusion to a stable, cohesive sense of who they are. To this end, they explore various opportunities within their social environments and eventually make commitments to certain options by identifying with specific values, goals, and roles. As part of the process of identity formation, adolescents also define themselves through their membership in social groups (Tajfel and Turner 1979), including a sense of belonging to national and supranational groups. European identity, as a form of supranational identity, has been shown to be a relevant identity dimension among adolescents living in Europe (Barrett 2007; Landberg et al. 2018) and to serve as a key foundation for popular support of the EU (Ciaglia et al. 2018; Hooghe and Marks 2004).

To gain a deeper understanding of identity development, recent process‐oriented models emphasize both the individual processes of identity formation and their specific constellations, which are referred to as identity status profiles (e.g., Crocetti et al. 2008; Luyckx et al. 2008). Building on Marcia's Identity Status Model (Marcia 1966), Crocetti et al. (Three Factor Model; 2008), for instance, distinguish three processes of identity formation and maintenance, including (1) commitment, (2) in‐depth exploration of how existing commitments align with one's goals, values and purposes as well as (3) reconsideration of existing commitments at the expense of more compelling alternatives. The combination of these processes results in five distinct identity statuses (profiles), each reflecting varying degrees of developmental maturity. A state of *diffusion* is characterized by low levels of all three processes. If a strong commitment is made without in‐depth exploration and reconsideration, individuals are considered to be in a state of *early closure*. A state characterized by high levels of reconsideration, without making or actively exploring commitments, is called *moratorium*. However, youth may have made commitments, but still actively explore in depth and reconsider them, reflecting a state of *searching moratorium*. Finally, identity formation reaches *achievement* when both commitment and in‐depth exploration are high, but existing commitments are stable without the need to reconsider them.

Research has shown that achievement profiles increase while diffusion profiles decrease from early adolescence to young adulthood, indicating that identity becomes more consolidated with age (Kroger et al. 2010). Furthermore, youth with more consolidated identities were found to exhibit more positive social and civic attitudes and behaviors (Crocetti et al. 2012). This is particularly true for individuals with an achieved identity status, which is considered the most mature and psychologically adaptive status. In contrast, moratorium and searching moratorium represent developmentally important but transitional phases, which may be accompanied by heightened distress, especially when a secure commitment is lacking, as in the case of moratorium. Youth with diffused or early closure profiles may feel content, as they do not experience a strong need for change; however, they often display lower levels of self‐reflection and civic engagement. In line with this assumption, research has shown that youth characterized by diffusion or early closure reported lower levels of civic engagement and prosocial behaviors compared to their achieved counterparts (e.g., Busch and Hofer 2011; Crocetti et al. 2011; Hardy and Kisling 2006; Lannegrand‐Willems et al. 2018).

While shared identification as Europeans has been shown to foster social and civic solidarity (e.g., tolerance and intergroup relations; Konings et al. 2023), status models have primarily been applied to interpersonal and role‐based domains of identity (e.g., partnership, friendship, education), and rarely to more distant facets of identity, such as regional (Borschel et al. 2019) and national identity (Greischel et al. 2019). Jugert and colleagues (2021) examined European and national identity status profiles among adolescents and young adults aged 15 to 26 years from Germany and the Czech Republic. Using a person‐oriented approach (Latent Profile and Latent Transition Analysis), they identified four European identity status profiles that could be interpreted as diffusion (GER: 13%; CZE: 11%), moratorium (GER: 36%; CZE: 23%), early closure (GER: 23%; CZE: 16%), and achievement (GER: 32%; CZE: 50%). The profiles showed moderate levels of stability over a 1‐year period, with achievement being most stable, and closure and diffusion the least stable. The age range of the sample, however, was too wide to draw conclusions about specific life stages, and potential age‐related factors that stimulate identity formation were not addressed.

## 2 The Formative Role of School Context

An important context for identity development in adolescence is the school environment (Lannegrand‐Willems and Bosma 2006). Not only do young people spend much time there, schools also share the common goal of educating young people to become open, tolerant, and mature citizens (Eckstein 2019). Research on school effects typically distinguishes formal, curricular factors, and non‐formal/informal learning experiences (Scheerens 2011). Regarding the former, Europe and the EU are typically included in secondary school curricula, providing important knowledge while also making Europe a potentially relevant topic for identity formation (The Standing Conference of the Ministers of Education and Cultural Affairs 2020; Ziemes et al. 2019).

Non‐formal and informal factors refer to relational, participatory, and structural characteristics, such as the prevailing school and classroom climate, opportunities for student participation, or experiences with diversity (Torney‐Purta 2002). School contexts that foster supportive teacher‐student relationships can provide a secure environment in which identity‐related questions are explored and reconsidered (Flum and Kaplan 2006). Opportunities for observational learning in such settings may further strengthen commitments and encourage in‐depth exploration (Rich and Schachter 2012). Supportive school environments also satisfy students' psychological needs (e.g., competence, autonomy, relatedness), thereby supporting identity formation (Madjar and Cohen‐Malayev 2013). Moreover, in schools young people from diverse backgrounds come together and accordingly affect each other's personal and social development (Miklikowska et al. 2021). This may promote the development of more sophisticated identity status by challenging existing commitments and encouraging reflection (Ceccon et al. 2024). Nevertheless, research systematically examining (European) identity profiles and their changes in relation to school experiences remains scarce.

## 3 The Present Study

The aim of the present study is twofold: (1) to shed light on the formation of European identity profiles, their civic‐ and solidarity‐related correlates, and changes over the course of one school year (Research Question 1), and (2) to examine the role of school‐based experiences in predicting European identity profiles and their changes over time (Research Question 2).

To this end, we used longitudinal data from German 9th graders collected in 2020/2021, thereby situating our study within a specific national context. As an early EU member state, Germany plays a central role within the EU, and EU‐related issues and discourses continue to feature prominently in the country's public debate. Cross‐national research has repeatedly shown that support for Europe and the EU is comparatively high among Germans, including young people (European Commission 2022; Matafora et al. 2024). At the same time, however, similar to trends in other European countries, German youth are increasingly susceptible to populist and anti‐EU‐related rhetoric (Schnetzer et al. 2024), which may undermine the development of a European identity.

Addressing the first research question, we applied Latent Profile Analysis (LPA) to the three identity formation processes *commitment*, *in‐depth exploration*, and *reconsideration*. We expected to replicate at least the four European identity status profiles identified by Jugert and colleagues (2021) and further hypothesized the emergence of a searching moratorium profile, as proposed by the Three‐Factor Model (Crocetti et al. 2008; RQ 1.1).

To validate the meaning and the social relevance of the identity profiles, we examined their associations with civic‐ and solidarity‐related correlates (i.e., EU‐related attitudes, tolerance, intentions for civic engagement; RQ 1.2). Both closure and achievement profiles are characterized by high levels of commitment. Accordingly, we expected adolescents in these profiles to exhibit more positive EU‐related attitudes. However, levels of tolerance and intentions for civic engagement were expected to be higher for youth in the achievement status as compared to closure and diffusion profiles (e.g., Crocetti et al. 2008; Lannegrand‐Willems et al. 2018). Concerning moratorium, we took an explorative approach as results may differ depending on commitment levels (i.e., moratorium vs. searching moratorium).

Next, we examined stability and change of profiles by applying Latent Transition Analysis (LTA; RQ 1.3). Given its transitional nature, we expected the moratorium profiles to be the least stable and the achievement status to be the most stable. Consistent with earlier findings (Kroger et al. 2010), we further expected to mainly find patterns of identity progression within the transition profiles (D → M; D → EC; D → A; EC → M; EC → A; M → A), while regressive transitions should be less likely.

To address the role of the school environment in European identity development (RQ 2), we finally examined how students' school experiences predict membership in European identity profiles and patterns of change. In particular, we expected that supportive relationships in school (e.g., student–teacher relationships) and an open school environment that values and supports diversity (e.g., a pluralistic learning climate) would promote both identity formation and identity consolidation (e.g., Ceccon et al. 2024; Rich and Schachter 2012). In other words, students who experience their school and class environment as more supportive and open should have a higher likelihood of being in either the mature achievement profile or the searching moratorium profile at the beginning of the school year (T1; RQ 2.1). In a similar vein, we expected that more positive school‐based experiences would be linked to identity progression (e.g., M → A; D → M/A; EC → M/A) over the course of one school year (RQ 2.2.).

## 4 Methods

### 4.1 Participants and Procedure

Data were drawn from a larger research project on youth and their attitudes towards the EU and Europe, conducted in two federal states of Germany (Thuringia and North Rhine‐Westphalia). The project was approved by the responsible institutional ethics committees, ensuring compliance with ethical standards. Paper‐pencil questionnaires were administered in 31 schools (90 classes) at the beginning and at the end of the 9th grade (school year 2021/2022; with permission of parents and school authorities). Overall, *N*_T1_ = 1,206 students participated at the first and *N*_T2_ = 1.096 at the second wave (86.7% response rate). At the beginning of the study (T1), students were on average *M* = 14.4 years old (SD = 0.6 years, range: 13–17 years), 52.1% identified as female (47.1% male; 0.8% diverse), and 25.1% reported having a migration background (i.e., at least one parent not born in Germany). Just over half of the students (59.5%) came from Thuringia, a federal state in the eastern part of Germany, characterized by rural areas and low ethnic diversity. The remaining students were from urban regions in North Rhine‐Westphalia, located in western Germany with a higher degree of ethnic diversity. Sample included students from 16 single‐track academic schools (58.3%), eight vocational schools (16.3%) and seven comprehensive schools (25.4%).

### 4.2 Measures

#### 4.2.1 European Identity

We used an adaptation of the Utrecht‐Management of Identity Commitments Scale (U‐MICS; Crocetti et al. 2010) that has already been tested and validated for regional and national identity domains (Greischel et al. 2019; Schubach et al. 2017). Dimensions of European identity were assessed with three items each: (1) *Commitment* (e.g., “I feel strong ties to Europe”; *ω*_1_ = 0.74, *ω*_2_ = 0.79), (2) *In‐depth Exploratio*n (e.g., “I often think about what it means to be European”; *ω*_1_ = 0.62, *ω*_2_ = 0.66), and (3) *Reconsideration* (e.g., “My feelings about Europe are changing”; all: 1 = *totally disagree*; 5 = *totally agree; ω*_1_ = 0.66, *ω*_2_ = 0.73). LPA analyses were based on the individual items. Due to a severely skewed distribution, one item of *In‐depth Exploratio*n was not suitable for later analyses and therefore had to be omitted.

#### 4.2.2 Civic‐ and Solidarity‐Related Correlates of Status Profiles

Students were asked to indicate how much they trust the EU (*EU Trust*; 1 = *not at all*; 5 = *completely*) and whether Germany should remain in the EU (*EU Support*; 1 = *definitely not*; 5 = *definitely*). Moreover, we assessed social and civic solidarity in terms of intentions for civic engagement and tolerance. For *Intentions for Civic Engagement*, students indicated how likely they would engage in activities such as taking part in a demonstration or signing a petition (10 items; *ω*_1_ = 0.83). *Tolerance* was assessed with four items reflecting students' negative attitudes toward refugees and immigrants (Gniewosz and Noack 2008; e.g., “Refugees and newly migrated people come here to exploit our welfare state”; 1 = *totally disagree*; 5 = *totally agree*; *ω*_1_ = 0.87). The scale was recoded so that higher scores reflect more tolerant attitudes.

#### 4.2.3 School Experiences

To assess *Student–Teacher‐Relationships*, we asked students to rate teachers' fairness, care, and respect on six items (Flanagan et al. 2007; e.g., “Most teachers take students' opinions seriously.”, 1 = *totally disagree*; 5 = *totally agree*; *ω*_1_ = 0.88). *Pluralistic Classroom Climate* was measured with six items of the Classroom Cultural Diversity Climate Scale (Schachner et al. 2021) at T1, encompassing three subdimensions: Intercultural Learning, Critical Consciousness, and Polyculturalism (*ω*_1_ = 0.79; for example, “At school we talk about how people can change when they meet people from other cultures.”; 1 = *totally disagree*; 5 = *totally agree*). Both scales were moderately correlated *r* = 0.31.

#### 4.2.4 Sociodemographic Correlates

*Age (in years)*, *Gender* (1 = *female* vs. 0 *= male and diverse*), and *Region* (1 = *North Rhine‐Westphalia* vs. 0 = *Thuringia*) were included as covariates. *School Type* was dummy coded with *vocational schools* (intermediate track, leading to vocational training or upper secondary school) and *comprehensive schools* (multitrack school, leading to vocational training, upper secondary school or university entrance qualification) being each compared to *single‐track academic schools* (leading to university entrance qualification).

### 4.3 Analytical Strategies

Analyses were conducted with M*plus* 8.7 (Muthén and Muthén 2017), using full information maximum likelihood (FIML) estimation for continuous variables. Standard errors were adjusted to account for clustering of the data at the classroom level (*Type = Complex* option).

To capture distinct European identity profiles (RQ 1.1), first, we conducted a Latent Profile Analysis (LPA) of the eight European identity items at T1. LPA, as a special form of Latent Class Analysis (LCA), is a probabilistic, person‐centered approach that identifies distinct groups of individuals based on their response patterns to continuous indicators while accounting for measurement error (Spurk et al. 2020). Models with two to six class solutions were estimated using multiple starting values. We compared the resulting class solutions based on general information criteria (AIC, BIC, sample size adjusted BIC), with lower values indicating better model fit (Tein et al. 2013). Furthermore, Bootstrap likelihood ratio test (BLRT) and Lo‐Mendell‐Rubin test (LMR test) were performed to test whether a model with *k* latent classes fits the data significantly better than a more parsimonious model with *k‐1* latent classes (Asparouhov and Muthén 2012). We further evaluated classification accuracy based on entropy (> 0.70) and average latent class probabilities (> 0.80). In addition, the number (parsimony) and interpretability of classes (i.e., inspection of mean levels of the three identity dimensions) were considered in selecting the optimal class solution. In a next step, we validated the class solution from the final model (RQ 1.2) by comparing the classes on social and civic solidarity correlates (i.e., EU‐related attitudes, tolerance, and intentions for civic engagement) and sociodemographic variables (via using BCH method for continuous correlates, Bakk and Vermunt 2016; DCATEGORICAL for categorical correlates, Lanza et al. 2013).

To examine whether the identity profiles persisted over time, we repeated the LPA at T2 (RQ 1.3). To ensure that profiles retained the same meaning across waves, measurement invariance was established by constraining within‐profile means and variances to be equal across T1 and T2. Further, as an extension of the LPA to repeated measurements, we conducted Latent Transition Analysis (LTA; Collins and Lanza 2010). LTA allows for the examination of stability and change in profiles by estimating transition probabilities between latent classes over time.

To address the role of the school environment (RQ 2), we added T1 *Student–Teacher‐Relationships* and *Pluralistic Classroom Climate* as predictors to the LTA models. This allowed us to test their effects on both the initial latent class probabilities at T1 and the latent transition probabilities over the school year. In addition, school type was included as a control variable for T1 probabilities.

## 5 Results

### 5.1 European Identity Profiles

#### 5.1.1 Types of Identity Profiles

Overall model comparisons and interpretability of identity profiles pointed to a four‐class solution (Table 1; see Supporting Information Table A for item‐level means). In detail, information criteria (AIC, BIC, aBIC) decreased with higher class solutions, but leveled off after four classes. BLRT consistently pointed to significant model improvements compared to more parsimonious models, but LMR test revealed no significant differences with five or more classes. Entropy and average latent class probabilities also indicated a reliable classification of students in the case of four identity profiles. Finally, when comparing class sizes and mean levels of the three identity dimensions for the four‐ and five‐profile solution, the fifth profile did not add substantial value ‐ neither theoretically (i.e., similar to profile four with high values on the all three identity domains) nor in terms of the number of classified students (approximately 6%; see Supporting Information Table B).

**Table 1:** Fit Indices for the LPA models with two to six class solutions at T1 and T2.

	No. of profiles	AIC	BIC	aBIC	Entropy	Average LC probabilities	*p* value LMR	*p* value BLRT
T1	2	27883.91	28011.18	27931.77	0.75	0.93/0.92	< 0.001	< 0.001
	3	27470.62	27643.71	27535.72	0.77	0.89/0.89/0.88	0.02	< 0.001
	4	27169.97	27388.88	27252.29	0.73	0.90/0.88/0.82/0.81	< 0.001	< 0.001
	5	27047.82	27312.55	27147.38	0.77	0.90/0.89/0.89/0.82/0.80	0.27	< 0.001
	6	26937.93	27248.48	27054.72	0.74	0.87/0.86/0.86/0.82/0.80/0.75	0.31	< 0.001
T2	2	25737.60	25862.43	25783.02	0.75	0.93/0.92	< 0.001	< 0.001
	3	25273.36	25443.13	25335.13	0.76	0.92/0.90/0.84	< 0.001	< 0.001
	4	24899.35	25114.05	24977.47	0.76	0.91/0.90/0.84/0.83	< 0.001	< 0.001
	5	24738.19	24997.82	24832.66	0.80	0.92/.90/0.87/0.85/0.83	0.08	< 0.001
	6	24558.00	24862.57	24668.82	0.82	0.92/0.90/0.87/0.87/0.85/0.84	0.02	< 0.001

*Note:* No. (number), AIC (Akaike Information Criterion), BIC (Bayesian Information Criterion), aBIC (Adjusted Bayesian Information Criterion), Average LC Probabilities (average latent class probabilities), LMR (Lo‐Mendell‐Rubin Likelihood Ratio Test), and BLRT (Bootstrap Likelihood Ratio Test).

**Figure 1:**
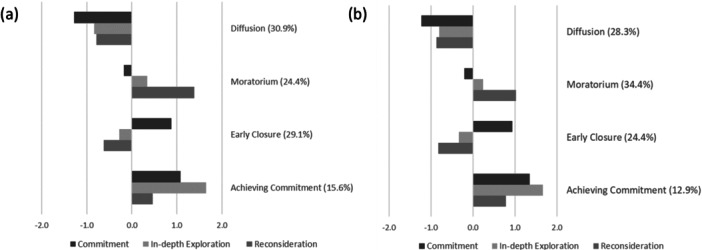
Profile patterns for commitment, in‐depth exploration, and reconsideration at T1 (a) and T2 (b), with the percentage of participants in each profile indicated in parentheses. For better interpretability and alignment with theoretical assumptions, the European identity items were combined into their original three subscales (z‐standardized scores).

Figure 1a shows the resulting four European identity profiles. The largest share of students (*n* = 371; 30.9%) showed a pattern of low scores on all three dimensions mirroring the *diffusion* status with low levels of commitment, in‐depth exploration, and reconsideration. A second large pattern (*n* = 350; 29.1%) was characterized by average to low levels of in‐depth exploration and reconsideration, but high levels of commitment, reflecting the status of *early closure*. The third profile replicated the idea of *moratorium* (*n* = 239; 24.4%), with average levels of commitment and in‐depth exploration and high levels of reconsideration. The last profile (*n* = 187; 15.6%) was characterized by high levels of commitment and in‐depth exploration, but also moderate to high levels of reconsideration. This pattern reflects aspects of both achievement and searching moratorium and, thus, is referred to it as *achieving commitment*.

#### 5.1.2 Civic‐ and Solidarity‐Related Correlates of Status Profiles

Membership in different status profiles was linked to civic‐ and solidarity‐related attitudes (Table 2). As expected, adolescents from the *early closure* and *achieving commitment* profile reported higher levels of EU trust and supported the idea that Germany should stay in the EU more strongly than adolescents from the *diffusion* and *moratorium* group. Adolescence from the *achieving commitment* group further reported more social and civic solidarity in terms of high intentions for civic engagement and high levels of tolerance. Intentions for civic engagement were further highest in the *moratorium* group. Contrary to our expectation, adolescence in the *diffusion* group also showed high social and civic solidarity (i.e., high levels of tolerance).

**Table 2:** Comparison of LPA profiles on sociodemographic and attitudinal correlates (T1) using univariate tests of equality of means and percentages with post hoc comparisons.

	Diffusion	Moratorium	Early closure	Achieving commitment	*χ* (3)	*p* value
*Sociodemographic correlates*						
% Female	51.5	52.8	49.9	56.0	1.29	0.730
% North Rhine‐Westphalia	44.3^a^	31.9^b^	40.0^a,b^	48.9^a^	11.40	0.010
Single‐track academic schools vs.						
% Vocational schools	21.6^a^	08.7^b^	20.4^a^	10.7^b^	22.41	< 0.001
% Comprehensive schools	26.4^a,b^	28.4^a,b^	18.9^a^	30.9^b^	8.20	0.042
Age (*M, SE*)	14.40 (0.04)	14.36 (0.05)	14.35 (0.04)	14.49 (0.06)	3.92	0.271
*Social and civic solidarity correlates*	*M(SE)*	*M(SE)*	*M(SE)*	*M(SE)*		
EU support	4.43 (0.05)^a^	4.41 (0.06)^a^	4.72 (0.04)^b^	4.77 (0.05)^b^	35.22	< 0.001
EU trust	2.87 (0.06)^a^	3.18 (0.06)^b^	3.40 (0.06)^c^	3.56 (0.07)^c^	68.71	< 0.001
Tolerance	3.85 (0.06)^a^	3.66 (0.08)^a,b^	3.51 (0.08)^b^	3.77 (0.09)^a^	11.74	0.008
Intentions for civic engagement	2.56 (0.05)^a^	2.99 (0.05)^b^	2.71 (0.05)^c^	3.14 (0.07)^b^	64.27	< 0.001

*Note:* Significant post hoc tests (*p* < 0.05) are indicated by different subscripts.

#### 5.1.3 Sociodemographic Correlates of Status Profiles

Comparisons of the identity profiles revealed no differences regarding age and gender, but significant variations by region and school type (Table 2). Adolescents from the western part of Germany (North Rhine‐Westphalia) were more likely to belong to the *diffusion* and *achieving commitment* profile, while adolescents from the eastern part were more represented in the *early closure* and *moratorium* profile. Further, adolescents from lower school tracks were overrepresented in the *diffusion* and *early closure* profile as compared to the other profiles.

### 5.2 Stability and Change of European Identity Profiles

Replicating the LPA at T2 yielded similar results to those obtained at the beginning of the school year. Measurement invariance of the profiles was established by constraining within‐profile means and variances to be equal across T1 and T2. The adjusted difference test (Satorra‐Bentler), based on log‐likelihood values and scaling factors, indicated a significant difference between the unrestricted and restricted models (*TRd*(40) = 76.82, *p* < 0.001). Nevertheless, the information criteria (AIC, BIC, aBIC) were lower for the more restricted model with measurement invariance, suggesting that despite the statistical difference, the invariant model is more parsimonious while maintaining a comparable fit (unrestricted model: AIC = 51,708.122, BIC = 52,195.937, aBIC = 51,894.173; restricted model: AIC = 51,699.414, BIC = 51,981.833, aBIC = 51,807.173).

Again, a four‐class solution was chosen based on model fit criteria (Table 1) and the interpretability and size of cluster solution (i.e., number of classified students for Cluster five < 5%; see Supporting Information Table B). Profile patterns, based on the mean levels of the three identity dimensions, remained consistent (Figure 1b), while the proportion of students in each profile changed slightly. At T2, most of the students belonged to the *moratorium* (*n* = 374; 34.4%) profile, followed by *diffusion* (*n* = 308; 28.3%) and *early closure* (*n* = 266; 24.4%). The *achieving commitment* profile remained the smallest group (*n* = 141; 12.9%).

Beyond the congruence of profile patterns, the results of the LTA provided evidence for both stability and change in identity patterns. Table 3 reports the latent transition probabilities, which indicate the likelihood of individuals remaining in the same identity status profile or moving to another profile between T1 and T2. The *diffusion* profile appeared to be the most stable, with 70.7% of students remaining in the same profile, followed by *early closure* (66.7%) and *moratorium* (61.2%). Unexpectedly, the *achieving commitment* profile was the least stable (48.9%). More than half of the students in this profile changed over the school year, with the majority transitioning to the *early closure* (23.6%) and *moratorium* (19.9%) profiles. Findings revealed both cases of identity regression, with transition probabilities between 0.08 (*achieving commitment* → *diffusion*) and 0.16 (*moratorium* → *diffusion*), as well as identity progression, with transition probabilities between 0.12 (*early closure* → *moratorium*) and 0.18 (*diffusion* → *moratorium*). The transition from *diffusion* to *achieving commitment* was least likely (0.02)

**Table 3:** Latent transition probabilities of identity status profiles from T1 to T2.

Identity profile T1	Identity profile T2			
	Diffusion	Moratorium	Early closure	Achieving commitment
Diffusion	0.707	0.178	0.093	0.022
Moratorium	0.162	0.612	0.104	0.126
Early closure	0.090	0.117	0.667	0.127
Achieving commitment	0.076	0.199	0.236	0.489

*Note:* Within‐profile means were constrained to be equal across T1 and T2.

### 5.3 The Role of School Experiences in Predicting European Identity Profiles

**Figure 2:**
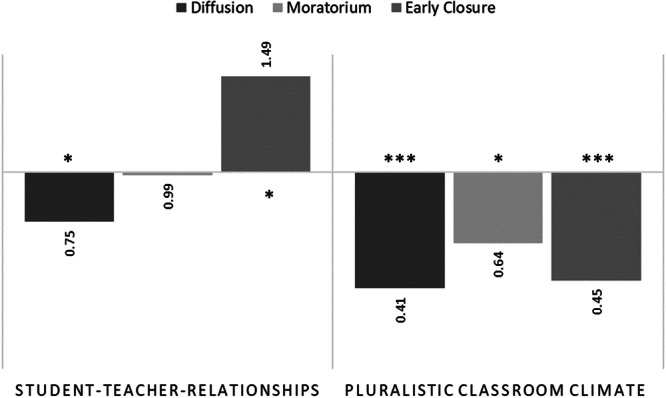
Odds ratios for school experiences as covariates in predicting identity class membership at Time 1 (reference group: achieving commitment). School track was included as a control variable. *p < 0.05, ***p < 0.001.

To examine the effects of school experiences, we extended the LTA analyses by including student–teacher‐relationships and pluralistic classroom climate at T1 as covariates, while controlling for school type. In line with our expectations, there were significant effects of school experiences on classification probabilities at T1. Figure 2 shows logistic regression odds ratios with *achieving commitment* as the reference group, indicating how the likelihood of belonging to a particular identity profile changes relative to the *achieving commitment* group (for descriptive purposes, unadjusted mean levels of school experiences across LPA profiles are provided in Supporting Information Table C). More favorable student–teacher‐relationships were associated with lower Odds of belonging to the profile of *diffusion* (OR = 0.748), but with higher Odds of belonging to the profile of *early closure* (OR = 1.489) at T1. Regarding the effects of a pluralistic classroom climate, adolescents from all identity profiles perceived their classroom climate as less pluralistic compared to those in the *achieving commitment* group.

Against our expectations, school experiences had hardly any effects on changes in identity profiles across time. The only exception was the effect of student–teacher relationships. In particular, perceiving more supportive teacher‐student relationships at T1 was associated with a lower likelihood of transitioning from early closure to moratorium. The remaining transition probabilities were not related to student–teacher relationships or classroom climate perceptions (Table 4).

**Table 4:** Odds ratios for school experiences as covariates in predicting transitions in identity class profiles.

	Teacher–Student‐Relationships			
	Identity profile T2			
Identity profile T1	Diffusion	Moratorium	Early closure	Achieving commitment
Diffusion	—	1.15	0.98	1.48
Moratorium	0.70	—	1.02	0.62
Early closure	0.89	0.46*	—	1.01
Achieving commitment	0.63	1.47	0.70	—

	Pluralistic classroom climate			
	Identity profile T2			
Identity profile T1	Diffusion	Moratorium	Early closure	Achieving commitment
Diffusion	—	1.05	0.68	1.40
Moratorium	1.16	—	0.81	1.06
Early closure	0.69	1.65	—	0.78
Achieving commitment	0.52	1.26	1.59	—

## 6 Discussion

The aim of the present study was to shed light on European identity formation among German adolescents over one school year (RQ 1), and to examine the role of school‐related experiences in predicting European identity profiles and profile transitions (RQ 2). The results of latent profile and latent transition (LPA, LTA) analyses revealed a pattern of four distinct status profiles that reflect varying degrees of identity consolidation and of civic and social solidarity. The findings further indicated that these profiles remained moderately to highly stable over the course of one school year. While most findings were in line with our expectations, three unexpected patterns emerged, which we discuss in greater detail in the following section: (i) we identified four main identity profiles, rather than the five theoretically anticipated; (ii) the *achieving commitment* profile showed relatively low stability over time; and (iii) although experiences in school were associated with identity profiles at the beginning of the school year, they did not predict transitions over time, except in one case.

Four distinct European identity profiles were identified among German 9th graders at the beginning of the school year, reflecting the statuses of diffusion, moratorium, early closure, and a form of achievement. While the first three profiles were consistent with theoretical classifications (e.g., Crocetti et al. 2008), and earlier findings on European identity formation (Author, 2021), the fourth profile showed unexpectedly high levels of reconsideration. This pattern diverges from conventional conceptualizations of achievement (in terms of high commitment and high in‐depth exploration, but low reconsideration), pointing to a dynamic of ongoing critical reassessment despite high commitment. Following Meeus et al. (1999), we refer to this pattern as *achieving commitment*.

This deviation in status patterns may reflect specific dynamics in the formation of European identity at this period in life. Youth at this age are just starting to engage more actively in political and civic topics, and, thus, their commitments are less firmly consolidated, making them more susceptible to change or abandonment (Van der Gaag et al. 2020). Furthermore, Europe, as an identity domain, is relatively abstract and distant, particularly in comparison to more immediate domains such as friendships or romantic relationships. As a result, it is more difficult for youth to establish a consolidated sense of European belonging, while, at the same time, there is also less environmental pressure to do so. Accordingly, “achieved” youth might remain more flexible in their commitments and choices (Kroger et al. 2010).

Despite the conceptual divergence, validation analyses confirmed that achieving commitment is characterized by greater maturity and serves as a source of solidarity. Students in this profile were more engaged and more tolerant, whereas youth with the early closure status reported less tolerant attitudes and lower intentions for civic engagement. These findings suggest that these young people may have simply adopted prevailing attitudes without much reflection, and that such unexamined commitments, however, have a limited capacity to translate into tolerant attitudes or active civic involvement. This relative “passivity” aligns with previous findings, showing that youth in the early closure status are less socially well‐adjusted than their achieved peers (e.g., lower levels of past and future volunteer engagement, social responsibility, and aspirations for community contribution; Crocetti et al. 2012). Students with a moratorium profile showed, in turn, high intentions for civic engagement, reflecting the “searching” nature of this state. Unexpectedly, youth from the diffusion profile appeared passive but tolerant. One possible explanation may lie in youth's understanding of being European (Mayer et al. 2025). Depending on whether this understanding is ancestry‐based (i.e., exclusive) or based on shared cultures and values (i.e., inclusive), it might relate differently to tolerance. Not identifying as European at all (or considering oneself cosmopolitan) may, in turn, prevent exclusive definitions and thereby foster intergroup relations and attitudes (Landberg et al. 2018).

About 16% of the adolescents were characterized by an elaborated European identity profile (achieving commitment), whereas approximately one‐third of the sample exhibited a diffusion profile. Although the proportions deviate from previous findings on European identity profiles in older samples (i.e., 32% achievement, 13% diffusion among German youth between 15 and 26 years; Jugert et al. 2021), they are consistent with estimates from other domains of identity for this age group (Kroger et al. 2010). Unexpectedly, the status of achieving commitment was not only the least common but also the least stable. While approximately 70% of students with an initial diffusion profile maintained their status over time, only about half of those initially classified as achieving commitment did so. This pattern underscores the fluid and dynamic nature of the *achieving commitment* profile, in contrast to conventional conceptualizations of achievement. As previously discussed, this may reflect adolescents' developmental stage, but it is most likely rooted in the abstract and distant character of the European identity domain. This, in turn, makes the development of consolidated commitments more difficult and may contribute to forms of identity regression. Specifically, some youth with an achieving commitment profile reduce their commitment and intensify their exploration over the course of the school year (i.e., transition to moratorium; 20%), whereas others revert to unreflective convictions and adopt a more rigid stance (i.e., transition to early closure; 26%).

Turning to the role of school experiences, the results confirmed that perceptions of the school context were associated with European identity profiles at the beginning of 9th grade. More precisely, a pluralistic classroom climate was related to more elaborated identity profiles (e.g., achieving commitment). Diverse learning opportunities and engagement with pluralistic cultural values seem to foster active exploration and commitment by challenging existing commitments and offering a variety of novel experiences. Contrary to our expectations, positive student–teacher relationships were associated with forms of early closure. Similar findings have been reported concerning the influence of parents and family on identity development, for example, in relation to secure attachment style (Årseth et al. 2009) or maternal caring behavior (Mullis et al. 2009). This suggests that supportive experiences foster commitment, whereas initiating identity exploration may require certain tensions or challenges that encourage young people to question existing (familiar) commitments and to engage in active exploration. In line with this explanation, more positive student–teacher relationships were also associated with a lower likelihood of transitioning from a status of early closure to a status of moratorium. However, remaining associations across time were not significant. One possible reason may be that the number of transitions was too small, resulting in insufficient statistical power to detect effects of school experiences on identity transitions.

### 6.1 Limitations and Future Directions

There are several limitations that should be addressed when interpreting the results. First, the study included only two measurement time points, one at the beginning and one at the end of the 9th grade. Although adolescence is an important period for identity development, the time span might be too short to identify long‐term changes in the formation of European identity. Moreover, more measurement occasions are needed to examine more differentiated developmental patterns such as alternating phases of identity regression and progression, or long‐term consolidation after an initial period of volatility. Second, there were important macro‐contextual events that needed to be taken into account. The study was conducted in 2020/2021, a time when the lives of youth were strongly impacted by the Covid‐19 pandemic. How Europe's or the EU's response to the pandemic was discussed in the media or at home during that time might have influenced the processes of European identity formation. For instance, especially at the onset of the pandemic, national strategies were mainly driven by self‐interest, manifested in border closures and controls, rather than a coordinated, solidarity‐based approach at the EU level. Public opinion was also divided regarding the EU's measures to address the pandemic (European Commission 2022). This could have impeded EU‐related identity formation processes. Moreover, disruptions to normal school life, such as temporary school lockdowns, remote learning, and classroom distancing, may have reduced the impact of school experiences at that time. Similarly, the study was conducted at the onset of the war in Ukraine, which may have substantially shaped youth's perceptions of Europe. Finally, our sample only included German adolescents. Recent election results have shown that populist parties are gaining votes in general, and particularly among young people (Schnetzer et al. 2024), which often includes anti‐EU and far‐right‐nativist narratives in the German context. Nevertheless, current surveys indicate that support for the EU and for remaining in the EU remains high in Germany (European Commission 2022). In line with this, the adolescents in our study expressed strong support for remaining in the EU. To determine whether the findings can be generalized to adolescents from other European countries, data from other countries are needed. Future studies should also examine in greater depth what European identity means for young people, particularly in terms of its potential to promote solidarity and strengthen intergroup relations (Mayer et al. 2055).

## 7 Conclusions

Overall, the study showed that distinct and theoretically‐based profiles of European identity can be identified in adolescence and that these profiles relate to different levels of civic and social solidarity. Despite a moderate stability of profile patterns, transitions over the course of one school year underline the developmental significance of the considered age group. Results further point to importance of contextual characteristics in European identity formation, such as school‐based experiences. In particular, a classroom climate that promotes diverse learning opportunities and embraces pluralistic values appeared to foster more elaborated identity profiles. In contrast, supportive experiences with teachers alone seem not to be sufficient, as they were linked with kinds of early closure.

## Ethics Statement

The study “Youth and Europe” was approved by the ethics committees of the University of Duisburg‐Essen and of the University of Jena (FSV 21/047).

## Conflicts of Interest

The authors declare no conflicts of interest.

## Supporting information

Table A *Mean levels and variances (in parentheses) of European identity profiles at T1 and T2*. Table B *Mean levels and variances (in parentheses) of European identity profiles at T1 and T2 for the five‐class solution*. Table C *Estimates of Mean Levels for School Experiences Across LPA Profiles at T1*.
