# Supplementary material for: European Identity in Youth: Longitudinal Dynamics and the Role of School Experiences
Source: J Adolesc. 2026 Jan 13;98(3):895–905. doi: 10.1002/jad.70097 (PMC13044864; doi:10.1002/jad.70097)
Supplement: Supplementary file 1 — Table A Mean levels and variances (in parentheses) of European identity profiles at T1 and T2. Table B Mean levels and variances (in parentheses) of European identity profiles at T1 and T2 for the five‐class solution. Table C Estimates of Mean Levels for School Experiences Across LPA Profiles at T1. [file JAD-98-895-s001.docx]

Table A

*Mean levels and variances (in parentheses) of European identity profiles at T1 and T2*

|  | | Diffusion | | Moratorium | | Early Closure | | Achieving Commitment | |
| --- | --- | --- | --- | --- | --- | --- | --- | --- | --- |
|  | | T1 | T2 | T1 | T2 | T1 | T2 | T1 | T2 |
| C1 | I feel strong ties to Europe. | 1.86 (0.80) | 1.87 (0.76) | 2.78 (0.80) | 2.80 (0.76) | 3.50 (0.80) | 3.69 (0.76) | 3.89 (0.80) | 4.09 (0.76) |
| C2 | I am proud to be European. | 2.30 (0.78) | 2.16 (0.75) | 3.23 (0.78) | 3.12 (0.75) | 4.00 (0.78) | 4.07 (0.75) | 4.18 (0.78) | 4.27 (0.75) |
| C3 | Being European gives me self-confidence. | 1.46 (0.92) | 1.50 (0.86) | 2.64 (0.92) | 2.57 (0.86) | 2.88 (0.92) | 3.07 (0.86) | 3.44 (0.92) | 3.88 (0.86) |
| E1 | I often think about what it means to be European. | 1.23 (0.41) | 1.24 (0.52) | 1.94 (0.41) | 2.01 (0.52) | 1.57 (0.41) | 1.63 (0.52) | 3.73 (0.41) | 3.60 (0.52) |
| E2 | I'm trying to get information about Europe. | 1.97 (1.24) | 2.14 (1.23) | 3.20 (1.24) | 3.16 (1.23) | 2.73 (1.24) | 2.67 (1.23) | 3.75 (1.24) | 3.87 (1.23) |
| R1 | My feelings about Europe are changing. | 1.63 (0.84) | 1.49 (0.78) | 3.20 (0.84) | 3.11 (0.78) | 1.95 (0.84) | 1.77 (0.78) | 2.98 (0.84) | 3.16 (0.78) |
| R2 | My idea of myself as a European is vague. | 1.66 (0.85) | 1.62 (0.92) | 2.69 (0.85) | 2.55 (0.92) | 1.86 (0.85) | 1.64 (0.92) | 2.14 (0.85) | 2.47 (0.92) |
| R3 | I think my understanding of what it means to be European might change in the near future. | 1.74 (1.01) | 1.51 (0.82) | 3.33 (1.01) | 3.11 (0.82) | 2.05 (1.01) | 1.74 (0.82) | 2.89 (1.01) | 3.10 (0.82) |

*Note*. C = Commitment, E = Exploration, R = Reconsideration.

Table B

*Mean levels and variances (in parentheses) of European identity profiles at T1 and T2 for the five-class solution*

|  | Cluster 1 | | Cluster 2 | | Cluster 3 | | Cluster 4 | | Cluster 5 | |
| --- | --- | --- | --- | --- | --- | --- | --- | --- | --- | --- |
|  | T1 | T2 | T1 | T2 | T1 | T2 | T1 | T2 | T1 | T2 |
|  | *n* = 367 (30.6%) | *n* = 325 (29.8%) | *n* = 189 (15.7%) | *n* = 410 (37.6%) | *n* = 371 (30.9%) | *n* = 188 (17.3 %) | *n* = 207 (17.2%) | *n* = 121 (11.1%) | *n* = 67 (5.6%) | *n* = 45 (4.0%) |
| C1 | 1.85 (0.76) | 1.89 (0.69) | 2.68 (0.76) | 3.07 (0.69) | 3.51 (0.76) | 3.82 (0.69) | 3.24 (0.76) | 4.21 (0.69) | 4.53 (0.76) | 1.83 (0.69) |
| C2 | 2.32 (0.81) | 2.21 (0.75) | 3.23 (0.81) | 3.34 (0.75) | 3.98 (0.81) | 4.19 (0.75) | 3.59 (0.81) | 4.35 (0.75) | 4.57 (0.81) | 2.55 (0.75) |
| C3 | 1.47 (0.89) | 1.53 (0.81) | 2.56 (0.89) | 2.84 (0.81) | 2.92 (0.89) | 3.12 (0.81) | 2.84 (0.89) | 3.96 (0.81) | 4.05 (0.89) | 1.50 (0.81) |
| E1 | 1.20 (0.28) | 1.24 (0.47) | 1.61 (0.28) | 2.16 (0.47) | 1.53 (0.28) | 1.53 (0.47) | 3.20 (0.28) | 3.78 (0.47) | 4.21 (0.28) | 1.37 (0.47) |
| E2 | 1.97 (1.24) | 2.17 (1.22) | 3.24 (1.24) | 3.22 (1.22) | 2.76 (1.24) | 2.58 (1.22) | 3.31 (1.24) | 3.93 (1.22) | 4.13 (1.24) | 2.75 (1.22) |
| R1 | 1.62 (0.85) | 1.50 (0.75) | 3.31 (0.85) | 2.91 (0.75) | 2.00 (0.85) | 1.64 (0.75) | 2.91 (0.85) | 3.16 (0.75) | 3.03 (0.85) | 3.94 (0.75) |
| R2 | 1.66 (0.85) | 1.63 (0.91) | 2.76 (0.85) | 3.40 (0.91) | 1.91 (0.85) | 1.55 (0.91) | 2.31 (0.85) | 2.43 (0.91) | 1.97 (0.85) | 3.02 (0.91) |
| R3 | 1.74 (01.03) | 1.52 (0.78) | 23.48 (01.03) | 2.88 (0.78) | 2.11 (01.03) | 1.62 (0.78) | 2.79 (01.03) | 3.08 (0.78) | 3.03 (01.03) | 4.07 (0.78) |

*Note*. C = Commitment, E = Exploration, R = Reconsideration.

Table C

*Estimates of Mean Levels for School Experiences Across LPA Profiles at T1*

|  | Diffusion | Moratorium | Early Closure | Achieving Commitment |
| --- | --- | --- | --- | --- |
|  | *M(SE)* | *M(SE)* | *M(SE)* | *M(SE)* |
| *Student-Teacher-Relationships* | 3.19 (.05) | 3.44 (.06) | 3.65 (.06) | 3.53 (.06) |
| *Pluralistic Classroom Climate* | 2.66 (.05) | 2.99 (.05) | 2.83 (.05) | 3.17 (.06) |

*Note*. Estimates based on BCH method for continuous variables
